# Effect of mean heart rate on 30-day mortality in ischemic stroke with atrial fibrillation: Data from the MIMIC-IV database

**DOI:** 10.3389/fneur.2022.1017849

**Published:** 2022-10-31

**Authors:** Shao-li Yao, Xi-wen Chen, Jie Liu, Xiao-rong Chen, Yao Zhou

**Affiliations:** ^1^Department of Neurology, Hospital of Chengdu Office of People's Government of Tibet Autonomous Region, Chengdu, China; ^2^Department of Neurology, The Second Affiliated Hospital of Chengdu Medical College, China National Nuclear Corporation 416 Hospital, Chengdu, China; ^3^Department of Vascular and Endovascular Surgery, Chinese PLA General Hospital, Beijing, China

**Keywords:** heart rate, mortality, ischemic stroke, atrial fibrillation, intensive care unit

## Abstract

**Background:**

The relationship of mean heart rate (MHR) with 30-day mortality in ischemic stroke patients with atrial fibrillation in the intensive care unit (ICU) remains unknown. This study aimed to investigate the association between MHR within 24 h of admission to the ICU and 30-day mortality among patients with atrial fibrillation and ischemic stroke.

**Methods:**

This retrospective cohort study used data on US adults from the Medical Information Mart for Intensive Care-IV (MIMIC-IV, version 1.0) database. Patients with ischemic stroke who had atrial fibrillation for and first time in ICU admission were identified from the MIMIC-IV database. We used multivariable Cox regression models, a restricted cubic spline model, and a two-piecewise Cox regression model to show the effect of the MHR within 24 h of ICU admission on 30-day mortality.

**Results:**

A total of 1403 patients with ischemic stroke and atrial fibrillation (mean [SD] age, 75.9 [11.4] years; mean [SD] heart rate, 83.8[16.1] bpm; 743 [53.0%] females) were included. A total of 212 (15.1%) patients died within 30 days after ICU admission. When MHR was assessed in tertials according to the 25th and 50th percentiles, the risk of 30-day mortality was higher in participants in group 1 (< 72 bpm; adjusted hazard ratio, 1.23; 95% CI, 0.79–1.91) and group 3 (≥82 bpm; adjusted hazard ratio, 1.77; 95% CI, 1.23–2.57) compared with those in group 2 (72–82 bpm). Consistently in the threshold analysis, for every 1-bpm increase in MHR, there was a 2.4% increase in 30-day mortality (adjusted HR, 1.024; 95% CI, 1.01–1.039) in those with MHR above 80 bpm. Based on these results, there was a J-shaped association between MHR and 30-day mortality in ischemic stroke patients with atrial fibrillation admitted to the ICU, with an inflection point at 80 bpm of MHR.

**Conclusion:**

In this retrospective cohort study, MHR within 24 h of admission was associated with 30-day mortality (nonlinear, J-shaped association) in patients with ischemic stroke and atrial fibrillation in the ICU, with an inflection point at about 80 bpm and a minimal risk observed at 72 to 81 bpm of MHR. This association was worthy of further investigation. If further confirmed, this association may provide a theoretical basis for formulating the target strategy of heart rate therapy for these patients.

## Introduction

Stroke is one of the major causes of death and disability in the world, which is characterized by a high incidence of morbidity, higher incidence of disability, high rate of mortality, high risk of recurrence, and high cost (1). Although new diagnostic and therapeutic techniques have emerged in the twenty-first century, such as functional brain imaging, cerebral perfusion imaging, intravenous thrombolysis, and mechanical thrombectomy, stroke is still a public health problem. Cardiogenic strokes, which make up 14% of all ischemic strokes (2), have quadrupled in the past few decades and, according to estimates from the United Kingdom, may triple once more by 2050 (3).

Previous studies have explored the relationship between heart rate and stroke outcomes, but each study used different heart rate parameters, such as baseline heart rate, mean heart rate, and heart rate variability (4–13). There is no consensus on which heart rate parameters or periods are best for the autonomic nervous system. Some studies employed heart rate parameters within a week after onset (14) and others within 24 h of admission (4, 8, 11, 15), while yet other studies used heart rate parameters at the time of the patient's first admission (6, 7, 16). Previous studies have reported controversial associations between heart rate and stroke outcomes. Studies have shown that high resting heart rates or low resting heart rates are associated with high mortality or future cardiovascular and cerebrovascular events (6, 7, 17). However, other studies have found that tachycardia and bradycardia do not independently predict the clinical course or outcome of stroke patients (18). The effect of heart rate variability on disease outcomes is also controversial (8–11, 13, 19).

However, most of the previous studies on the relationship between heart rate and prognosis of ischemic stroke have been conducted in patients with mild to moderate stroke, and the definition of heart rate parameters varies from study to study, and most of them focus on the relationship between heart rate parameters and medium- and long-term prognosis. As it has an erratic rhythm, atrial fibrillation (AF), which is the most frequent reason for heart thrombus development and is to blame for 45% of cardiogenic strokes (20), has often been excluded. The association between mean heart rate (MHR) and stroke prognosis in patients with atrial fibrillation is uncertain because most studies did not include patients with AF. Therefore, this study aimed to investigate the association between MHR and 30-day mortality in patients with ischemic stroke and AF admitted to the intensive care unit (ICU).

## Materials and methods

### Study population

This retrospective cohort study used the Medical Information Mart for Intensive Care-IV (MIMIC-IV version 1. 0) database (21, 22). This is a longitudinal, single-center database that contains data from 2008 to 2019. The overall information was saved as a relational database, consisting of patient demographics, vital signs, laboratory tests, diagnostic information, treatment information, and in-hospital mortality. One author (Shaoli Yao, ID: 10808597) who has finished the Collaborative Institutional Training Initiative examination can access the database and was responsible for data extraction and analysis. The use of the MIMIC-IV database was approved by the review boards of the Massachusetts Institute of Technology and Beth Israel Deaconess Medical Center. The data are anonymous, and the requirement for informed consent was therefore waived. The code of data extraction is available on GitHub (23) (http://github.com/MIT-LCP/mimic-iv). All reporting followed the Strengthening the Reporting of Observational Studies in Epidemiology guidelines (24). The study was conducted in accordance with the Declaration of Helsinki (as revised in 2013).

Patients with ischemic stroke who had AF for and first time in ICU admission were considered eligible for our study. The diagnosis of ischemic stroke and AF were based on the International Classification of Disease, the Ninth Version, and the Tenth Version (Supplementary Table 1). As ischemic stroke may not always be listed as the principal diagnosis, we also included records with ischemic stroke in any of the first five diagnostic positions according to the diagnostic sequence. The inclusion criteria were as follows: (1) patients were aged ≥18 years; (2) patients were in ICU for more than 24 h; and (3) only the first ICU admission was considered. The exclusion criteria were as follows: (1) patients were aged < 18 years; (2) patients had a minimum heart rate < 35 beats per minute; (3) heart rate data were not available; and (4) patients were in ICU for < 24 h.

### General data collection

The Structured Query Language was used for data extraction (23). All vital signs, Sequential Organ Failure Assessment (SOFA) score, and Simplified Acute Physiology Score II (SAPS II) were collected within 24 h of admission to ICU. We extracted the following variables: (1) basic demographics, including age, sex, weight, insurance, marital status, and ethnicity; (2) vital signs and the severity of illness, which was defined at ICU admission using the SOFA score, SAPS II, Glasgow Coma Scale (GCS), and Charlson comorbidity index; (3) treatment, including ventilation use, vasoactive drug use, and dialysis use; and (4) comorbidities, myocardial infarct, congestive heart failure, cerebrovascular disease, dementia, chronic pulmonary disease, renal disease, cancer, severe liver disease, and metastatic solid tumor. Height and body mass index were not included because more than 50% of the data were missing in this study.

### Variable definition and outcomes

Tracheotomy, invasive ventilation, and noninvasive ventilation were all considered to be indications of ventilation use in patients. Vasoactive drugs included norepinephrine, epinephrine, phenylephrine, vasopressin, dopamine, dobutamine, isoprenaline, sodium nitroprusside, nicardipine, labetalol, esmolol, and diltiazem. The MHR was defined as the calculated average (by adding together all of the heart rate readings recorded and dividing by the total number of readings) of the heart rate measured within 24 h of admission to the ICU. The mean (SD) of the heart rate recordings was 28.9 (8.4). According to the 25th and 50th percentiles of MHR, participants were divided into three groups. Cardiovascular disease (CVD) is defined as a history of myocardial infarction or congestive heart failure. In this study, we regarded 30-day mortality as the outcome event, which was also extracted from the MIMIC-IV database. The outcome events were monitored up to 30 days after admission to ICU.

### Statistical analysis

The distribution of the baseline data of the patients included in this study was presented for the different outcome groups. Categorical data were presented as a number (percentages), while continuous data were presented as the mean ± standard deviation or median (interquartile range), as appropriate. Differences in continuous variables were tested using the analysis of variance test or rank-sum test as appropriate. The chi-square test or Fisher's exact test for categorical variables was applied to compare the characteristics of the study subjects among the outcome groups.

We simply replaced the missing data with a median because 5% of the GCS score was missing. Because the percentage of missing data was small (missing rate varied from 0.5 to 0.7%) for mean glucose and weight, no imputation method was used. Multivariable Cox regression analyses were performed to assess the independent association between MHR and 30-day mortality. Mean heart rate was entered as a categorical variable (tertials) and as a continuous variable (with a hazard ratio (HR) calculated per 10 bpm MHR increase). We applied four models in the regression analysis. Multivariable models were adjusted as follows: model 1 was not adjusted; model 2 was adjusted for age, gender, mean blood oxygen saturation (spo2), mean glucose, weight, and Charlson's comorbidity index; model 3 was adjusted for model 2 plus SOFA score, SAPS II, and GCS; and model 4 was adjusted for model 3 plus ventilation use and vasoactive drug use. Survival curves were plotted by Kaplan–Meier and log-rank analyses.

We used restricted cubic spline models to examine the possible nonlinear association between the levels of MHR and the incidence of 30-day mortality (25). Analyses treating MHR levels of 80 bpm as the reference with adjustment of the aforementioned (model 4) covariates, and a knot was located at the 5th, 35th, 65th, and 95th percentiles of the MHR. Threshold analysis in the association of MHR with the study outcome was conducted with a likelihood ratio test comparing the model with only a linear term against the model with linear and cubic spline terms. We considered that the association between MHR level and 30-day mortality may be influenced by ventilation use, vasoactive drug use, history of cardiovascular diseases, etc. Therefore, heterogeneity across subgroups was assessed by Cox proportional hazards models, and interactions between subgroups were examined by likelihood ratio testing.

As we included patients with ischemic stroke in any of the first five diagnostic positions by the diagnostic sequence and could not rule out the possibility that some patients were admitted to the ICU for other illnesses, we included patients with ischemic stroke as the first diagnosis in the second dataset. The analysis described above was then carried out in the second dataset to ensure the reliability and validity of our findings.

A two-tailed test was performed, and a P < 0.05 was considered statistically significant in our study. All the analyses were performed with the statistical software packages R (http://www.R-project.org, The R Foundation) and Free Statistics software version 1.6 (26).

## Results

### Baseline characteristics of the study patients

Three thousand and nine hundred and eight individuals with ischemic stroke who were admitted to the ICU for the first time were identified according to the International Classification of Disease, the Ninth Version, and the Tenth Version. Among them, 3,715 individuals with ischemic stroke were listed as the first five diagnostic positions according to the diagnostic sequence, and 1,635 individuals had AF. After screening according to the exclusion criteria, the final cohort included 1,403 patients with ischemic stroke and AF. Of these patients, 212 (15.1%) died within 30 days after ICU admission. The detailed flowchart of participant recruitment is shown in Supplementary Figure 1. The interested reader can find them in Supplementary material online.

The mean age of the 1403 patients was 75.9 ± 11.4 years, and about half of them were female (53.0%). The baseline characteristics of the population included in the study are listed in Table 1. When compared with the survivors, the heart rate parameters, SOFA score, and SAPS II were higher in deceased patients. The deceased patients were more likely to be older and combined with many other diseases compared with the survivors.

**Table 1:** Baseline characteristics of patients stratified by 30-day mortality.

**Variables**	**Total** **(*n* = 1403)**	**Survivors** **(*n* = 1191)**	**Non-survivor**s **(*n* = 212)**	***p* value**
ICU stay, day	8.7 (5.0, 15.1)	9.1 (5.3, 15.7)	6.2 (3.1, 11.7)	< 0.001
Minimum HR, bpm	67.8 ± 14.5	67.3 ± 14.2	70.8 ± 15.8	0.001
Maximum HR, bpm	105.2 ± 23.4	103.8 ± 22.9	112.6 ± 24.9	< 0.001
MHR, bpm	83.8 ± 16.1	83.0 ± 15.7	88.4 ± 17.9	< 0.001
HRF, bpm	33.0 (24.0, 46.0)	31.0 (23.0, 44.0)	39.0 (27.0, 53.2)	< 0.001
MBP, bpm	82.9 ± 12.0	82.9 ± 11.9	82.9 ± 12.5	0.950
Mean respiratory rate, times/min	19.5 ± 3.4	19.3 ± 3.2	20.8 ± 4.1	< 0.001
Mean body temperature, °C	36.8 ± 0.5	36.8 ± 0.4	36.9 ± 0.7	0.194
Mean spo2, %	97.2 (95.8, 98.4)	97.1 (95.7, 98.3)	97.9 (96.1, 99.1)	< 0.001
Mean blood glucose, mg/dl	140.8 ± 42.9	138.9 ± 41.4	152.0 ± 49.3	< 0.001
Sofa score	4.0 (3.0, 7.0)	4.0 (2.0, 6.0)	6.0 (4.0, 8.0)	< 0.001
Weight, kg	79.3 ± 21.3	80.2 ± 21.3	74.5 ± 20.7	< 0.001
SAPSII	38.3 ± 11.6	37.0 ± 10.7	45.6 ± 13.6	< 0.001
Age, years	75.9 ± 11.4	75.3 ± 11.6	79.3 ± 10.0	< 0.001
Charlson's comorbidity index	7.5 ± 2.4	7.3 ± 2.3	8.3 ± 2.3	< 0.001
GCS	13.0 (8.0, 14.0)	13.0 (9.0, 14.0)	7.0 (4.0, 10.0)	< 0.001
Gender, *n* (%)				0.146
Female	743 (53.0)	621 (52.1)	122 (57.5)	
Male	660 (47.0)	570 (47.9)	90 (42.5)	
Myocardial infarct, *n* (%)				0.107
NO	1135 (80.9)	972 (81.6)	163 (76.9)	
Yes	268 (19.1)	219 (18.4)	49 (23.1)	
Congestive heart failure, *n* (%)				0.059
NO	856 (61.0)	739 (62)	117 (55.2)	
Yes	547 (39.0)	452 (38)	95 (44.8)	
Cerebrovascular disease, *n* (%)				< 0.001
NO	356 (25.4)	340 (28.5)	16 (7.5)	
Yes	1047 (74.6)	851 (71.5)	196 (92.5)	
Dementia, *n* (%)				0.134
NO	1316 (93.8)	1122 (94.2)	194 (91.5)	
Yes	87 (6.2)	69 (5.8)	18 (8.5)	
Chronic pulmonary disease, *n* (%)				0.835
NO	1093 (77.9)	929 (78)	164 (77.4)	
Yes	310 (22.1)	262 (22)	48 (22.6)	
Renal disease, *n* (%)				0.323
NO	1057 (75.3)	903 (75.8)	154 (72.6)	
Yes	346 (24.7)	288 (24.2)	58 (27.4)	
Malignant cancer, *n* (%)				0.207
NO	1294 (92.2)	1103 (92.6)	191 (90.1)	
Yes	109 (7.8)	88 (7.4)	21 (9.9)	
Severe liver disease, *n* (%)				0.309
NO	1386 (98.8)	1178 (98.9)	208 (98.1)	
Yes	17 (1.2)	13 (1.1)	4 (1.9)	
Metastatic solid tumor, *n* (%)				0.063
NO	1359 (96.9)	1158 (97.2)	201 (94.8)	
Yes	44 (3.1)	33 (2.8)	11 (5.2)	
Dialysis				0.101
No	1335 (95.2)	1138 (95.5)	197 (92.9)	
Yes	68 (4.8)	53 (4.5)	15 (7.1)	
Ventilation, *n* (%)				< 0.001
No	907 (64.6)	813 (68.3)	94 (44.3)	
Yes	496 (35.4)	378 (31.7)	118 (55.7)	
Vasoactive drugs, *n* (%)				< 0.001
No	646 (46.0)	572 (48)	74 (34.9)	
Yes	757 (54.0)	619 (52)	138 (65.1)	
Insurance				0.445
Medicaid	59 (4.2)	51 (4.3)	8 (3.8)	
Medicare	838 (59.7)	703 (59)	135 (63.7)	
Other	506 (36.1)	437 (36.7)	69 (32.5)	
Marital status, *n* (%)				0.148
Divorced	88 (7.1)	79 (7.3)	9 (5.3)	
Married	605 (48.6)	530 (49.2)	75 (44.4)	
Single	254 (20.4)	221 (20.5)	33 (19.5)	
Widowed	299 (24.0)	247 (22.9)	52 (30.8)	
Ethnicity, *n* (%)				< 0.001
American Indian/Alaska Native	3 (0.2)	3 (0.3)	0 (0)	
Asian	54 (3.8)	47 (3.9)	7 (3.3)	
Black/African-American	137 (9.8)	116 (9.7)	21 (9.9)	
Hispanic/Latino	34 (2.4)	29 (2.4)	5 (2.4)	
Other	50 (3.6)	43 (3.6)	7 (3.3)	
Unable to obtain	12 (0.9)	8 (0.7)	4 (1.9)	
Unknown	180 (12.8)	126 (10.6)	54 (25.5)	
White	933 (66.5)	819 (68.8)	114 (53.8)	

For each variable, mean ± standard deviation, median (interquartile range), or number (percent) was reported (as appropriate).

ICU, Intensive care unit; MHR, mean heart rate; bpm, beats per minute; HRF, Heart rate fluctuation; MBP, mean blood pressure; SpO2, blood oxygen saturation; SOFA, sequential organ failure assessment; SPASII, simplified acute physiology score II; GCS, glasgow coma scale.

### Effects of MHR on 30-day mortality

**Figure 1:**
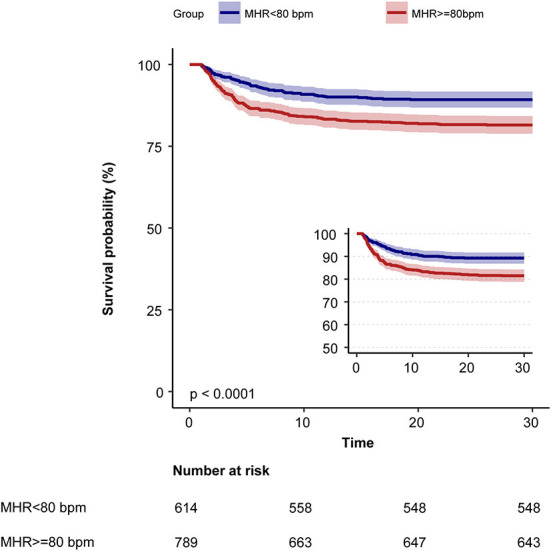
Kaplan–Meier survival curves for day 30 of ischemic stroke patients with atrial fibrillation. MHR, mean heart rate; bpm, beats per minute.

**Figure 2:**
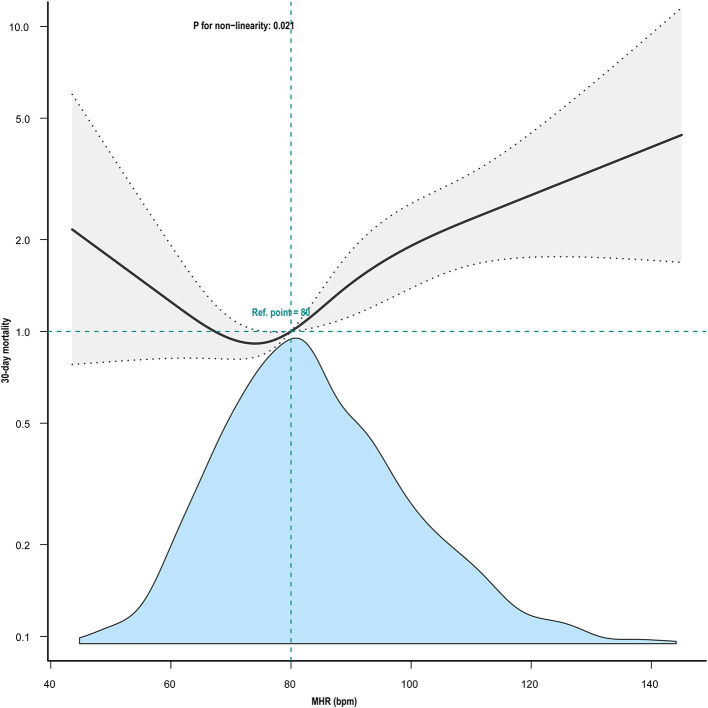
Nonlinear association between mean heart rate and 30-day mortality. Adjustment factors included gender, mean SpO2, mean glucose, weight, SOFA score, Charlson's comorbidity index, SAPS II, age, GCS, Ventilation use, and vasoactive drug use. The black line and gray area represent the estimated values and their corresponding 95% confidence intervals, respectively. MHR, mean heart rate; bpm, beats per minute.

Kaplan–Meier curve showed there was lower mortality by day 30 in patients with MHR < 80 bpm (log-rank test: *p* < 0.0001, Figure 1). In the multivariable Cox models (Table 2), we observed that the risk of 30-day mortality was higher in participants in group 1 (< 72 bpm; adjusted HR, 1.23; 95% CI, 0.79–1.91) and group 3 (≥82 bpm; adjusted HR, 1.77; 95% CI, 1.23–2.57) compared with those in group 2 (72–81 bpm). After adjustment for confounding factors, a 19% higher 30-day mortality could be shown in patients with MHR increased per 10 bpm. Multivariable-adjusted restricted cubic spline analyses suggested J-shaped associations of MHR with 30-day mortality (Figure 2, *p* = 0.021).

**Table 2:** Hazard ratio and 95% CI of mean heart rate for 30-day mortality.

**Variable**	**Model 1**		**Model 2** ^**†**^		**Model 3** ^**†**^		**Model 4** ^**†**^	
	**HR (95%CI)**	***P* value**	**HR (95%CI)**	***P* value**	**HR (95%CI)**	***P* value**	**HR (95%CI)**	***p* value**
MHR per10, bpm	1.2 (1.11–1.29)	< 0.001	1.21 (1.11–1.32)	< 0.001	1.16 (1.07–1.27)	< 0.001	1.19 (1.09–1.3)	< 0.001
MHR tertials, bpm								
< 72	1.21 (0.78–1.87)	0.403	1.22 (0.79–1.89)	0.378	1.2 (0.77–1.87)	0.416	1.23 (0.79–1.91)	0.363
72–81	1(Reference)		1(Reference)		1(Reference)		1(Reference)	
≥82	1.92 (1.34–2.76)	< 0.001	1.91 (1.32–2.75)	0.001	1.71 (1.18–2.46)	0.004	1.77 (1.23–2.57)	0.002
P for trend		0.001		0.002		0.015		0.012

MHR, mean heart rate; bpm, beats per minute, HR, hazard ratio; CI, Confidence interval.

† There were 9 patients missing blood glucose data and 10 patients missing weight data, using sample size = 1384.

Model 1: no adjusted.

Model 2: adjusted for gender, mean SpO2, mean glucose, weight, Charlson's comorbidity index, and age.

Model 3: adjusted for model 2 plus SOFA score, SAPS II, and GCS.

Model 4: adjusted for model 3 plus ventilation use, and vasoactive drugs use.

Using a two-piecewise Cox regression model, we found that the threshold of MHR was 80 bpm (Table 3). Above the threshold, for every 1-bpm increase in MHR, there was a 2.4% increase in 30-day mortality (adjusted HR, 1.024; 95% CI, 1.01–1.039) (Table 3).

**Table 3:** Threshold analyses of MHR on 30-day mortality using two-piecewise regression models.

**Threshold of MHR, bpm**	**HR**	**95% CI**	***P* value**
< 80	0.974	0.943–1.006	0.1117
≥80	1.024	1.01–1.039	< 0.001
Likelihood ratio test	–	–	0.0070

MHR, mean heart rate; bpm, beats per minute; HR, hazard ratio; CI, confidence interval.

Adjustment factors included gender, mean SpO2, mean glucose, weight, SOFA score, Charlson's comorbidity index, SAPS II, age, GCS, ventilation use, vasoactive drugs use.

### Sensitivity analysis

Much more sensitivity analyses were run than can be included in the article. The interested reader can find them in Supplementary material online. After subgroup analysis according to the confounders including age, gender, SOFA score, CVD disease, ventilation use, and vasoactive drug use (Supplementary Figure 2), the result remains robust, and we did not observe any significant interaction in the subgroups (all *p*-values for interaction > 0.05).

Because GCS data for 74 individuals were not available, they were excluded from the sensitivity analysis. The association between MHR and 30-day mortality has remained steady (Supplementary Table 2).

In patients with ischemic stroke as the first diagnosis, we also observed that the association between MHR and 30-day mortality was consistently significant in all models in the multivariable Cox models (Supplementary Table 3). Kaplan–Meier curve also showed there was lower mortality by day 30 in patients with MHR < 80 bpm (log-rank test: *p* < 0.001, Supplementary Figure 3).

## Discussion

### The main result

This study aimed to analyze the relationship between MHR and short-term outcomes in patients with ischemic stroke and AF admitted to the ICU. MHR within 24 h after admission to ICU was found to be independently associated with 30-day mortality. Furthermore, a typical J-shaped curve was observed in restricted cubic splines for the association between MHR and 30-day mortality in our study population, indicating an inflection point at about 80 bpm and minimal risk observed at 72 to 81 bpm of MHR.

### Effects of heart rate parameters on 30-day mortality

Numerous studies have found that heart rate was associated with the prognosis of many diseases, including coronary heart disease, myocardial infarction, heart failure, acute ischemic stroke, acute hemorrhagic stroke, and so on (7, 9, 11, 13, 27–30). In addition, heart rate variability is associated with the incidence and duration of poststroke depression (27). In patients with AF, the association between MHR and short-term prognosis in stroke patients has been limited. Lee et al. found a J-shaped association between MHR and 1-year mortality after stroke, with an optimal mean HR of about 80 bpm (13). This was in line with our findings. Our restricted cubic splines clearly showed a J-shaped curve for the association between MHR and the 30-day mortality in our study population. Kaplan–Meier curve showed there was lower mortality by day 30 in patients with mean HR < 80 bpm. Interestingly, for the 30-day mortality of patients with ischemic stroke and AF admitted to the ICU, the lowest risk of MHR was ~80 bpm, which might be a candidate marker for decision making in HR control strategies. However, an observational study carried out by Steinberg Ba et al. showed a J-shaped relationship between heart rate and mortality in patients with permanent AF, and a heart rate around 65 bpm seems to be the optimal heart rate (5). Meanwhile, Böhm et al. found a nonlinear relationship between MHR and stroke incidence in patients with diabetes, with the lowest risk of stroke at an MHR of 65 bpm (31). This difference indicates that the optimum heart rate may differ among populations.

In patients with AF, the small number of studies on the relationship between heart rate and prognosis in stroke patients is controversial. However, Han et al. found a different result. In their study, no independent association between heart rate and in-hospital mortality was observed in patients with acute ischemic stroke who had AF (16). We speculate that the reasons for our inconsistent findings may be as follows: The study conducted by Han et al. used heart rate at admission; as a result of arrhythmia in patients with AF, only one heart rate measurement may not be representative; on the contrary, their study population was mainly concentrated in patients with minor stroke (median National Institutes of Health Stroke Scale (NIHSS) score was 4.0).

The pathophysiological mechanism of cardiovascular autonomic dysfunction in patients with ischemic stroke remains unclear, and we speculate that the following mechanisms may be involved. There was increasing evidence suggesting that the pathophysiological process of acute stroke is not an isolated brain process. Inflammatory, endocrine, and autonomic pathways are activated simultaneously with the ischemic cascade of systemic responses (32, 33). After a stroke, activation of the sympathetic nervous system is thought to be a trigger for systemic immunodepression and an increased risk of infection, which is also one of the major risk factors for mortality and disability (34). A fast heart rate may also indicate sympathetic nerve overactivity, which has been related to inflammatory processes and higher blood pressure at night, both of which are well-known indicators of stroke mortality (35–37). Previous studies on the relationship between heart rate variability and stroke outcomes have also found that patients with low heart rate variability have a worse prognosis, while low heart rate variability indicates high sympathetic nervous system activity (8, 10, 11). In addition to the increased sympathetic nervous system tone in the acute phase of stroke, Lee et al. found a stronger association between mean heart rate and mortality in the late acute stage (13). Autonomic nervous system dysfunction after stroke exacerbated subsequent brain damage *via* changes in hemodynamics and non-hemodynamic variables (38). Higher or lower heart rate in the acute phase of a stroke may lead to a reduction in cardiac output resulting in insufficient perfusion of the ischemic area and ultimately adverse outcomes.

### Strengths and limitations

Our study has some strengths. First, to the best of our knowledge, the association of heart rate with short-term prognosis has not been developed in patients with ischemic stroke and AF admitted to the ICU, and our study found a J-shaped association between MHR and 30-day mortality. The lowest risk of death was found when the MHR was about 80 bpm, which may provide a theoretical basis for formulating the target strategy of heart rate therapy for these patients. This finding extends conclusion to a wider range of clinical entities. Second, we adopted the MHR, which is easy to get and easy for clinicians to use. Third, we performed multiple sensitivity analyses: (1) the use of vasoactive drugs may have an effect on heart rate in ICU, acute stroke patients were exposed to varying degrees of artificial light, noise, and various organ support, which usually leads to dysrhythmias in sleep architecture, blood pressure, and HR (39), so we performed subgroup analyses by age, sex, SOFA score, vasoactive drugs use, ventilation use, and the results remained stable; (2) we used multi-model adjustment in Cox regression analyses to correct for the effect of confounders, which remained stable after full model adjustment (model 4); (3) the MHR was analyzed with continuous and categorical variables in the regression model and this method can reduce the chance of data analysis and improve the stability of the results; and (4) according to the diagnostic sequence, the patients with ischemic stroke ranked in the first five or the first were analyzed, and the results were still stable.

This study has some limitations. First, previous studies have found that heart rate variability (HRV) as an autonomous cardiac biomarker is associated with prognosis in a variety of diseases, and its calculation methods can be divided into linear and nonlinear methods with the high cost and low clinical availability, and we did not use this parameter because we could not get the corresponding data in MIMIC-IV database to calculate HRV in the time domain and frequency domain. However, our study is based on real-world clinical data, and heart rate parameters are measured at the bedside, which are simple and easy to obtain and use by a clinician at the bedside. Second, it is well known that the NIHSS score is widely used to assess the severity of the ischemic stroke, and previous studies have found that the NIHSS score is an independent predictor of stroke outcome (9), but because these data were unavailable in the MIMIC-IV database, we could not include this variable for analysis. However, we included the GCS score, another scoring system for assessing neurological function, and in the regression model, MHR remained positively associated with 30-day mortality in our study population after adjustment for the GCS score. Third, while caution should be used when extending the results because the study population was restricted to a single nation (the USA) and a single ICU institution, our sample size was sizable and relatively representative. Future multicenter prospective studies may be done to confirm our findings. Fourth, many factors can affect the prognosis of stroke patients, such as the strategy of reperfusion therapy in the acute phase. We could not exclude the effects of this factor on our result as the data were not accessible in the MIMIC-IV database, but we attempted to adjust for the effect of available confounders. Our results are consistent with the conclusion of a multicenter prospective cohort study conducted by Lee et al., which included a reperfusion therapy strategy for adjustment (13). Fifth, selection bias is inevitable due to the design of retrospective cohort studies, and future randomized controlled trials would help confirm our findings. Sixth, the patient's condition at the moment of the heart rate measurement was not recorded in the MIMIC-IV1.0 database. And this may affect the real relationship between mean heart rate and 30-day mortality in ischemic stroke with atrial fibrillation. These associations were worthy of further investigation. Seventh, studies have indicated a connection between dysautonomia and certain arrhythmia patterns and different parts of the central nervous system (40–42). Unfortunately, the MIMIC-IV database does not have information on the location and sizes of the strokes. Future prospective studies may further investigate the impact of various lesion sites and sizes on heart rate parameters and in-hospital all-cause mortality.

## Conclusion

In conclusion, this retrospective cohort study revealed a J-shaped association between MHR within 24 h of admission and 30-day mortality in patients with ischemic stroke and atrial fibrillation in the ICU, with increased 30-day mortality when MHR > 80 bpm. This association was worthy of further investigation. If further confirmed, this association may provide a theoretical basis for formulating the target strategy of heart rate therapy for these patients.

## Data availability statement

The data analyzed in this study was obtained from the Medical Information Mart for Intensive Care IV (MIMIC-IV) Clinical Database, the following licenses/restrictions apply: To access the data you must be a credentialed user, complete the required training (CITI Data or Specimens Only Research) and sign the data use agreement for the project. Requests to access these datasets should be directed to PhysioNet, https://physionet.org/; https://doi.org/10.13026/s6n6-xd98.

## Ethics statement

The studies involving human participants were reviewed and approved by the review boards of the Massachusetts Institute of Technology and Beth Israel Deaconess Medical Center. Written informed consent for participation was not required for this study in accordance with the national legislation and the institutional requirements.

## Author contributions

S-lY participated in the design of research schemes, extracted and analyzed the data, and wrote the main manuscript text. X-wC collated the data. YZ and X-rC participated in the design of research schemes. JL reviewed the manuscript. All authors contributed to the article and approved the submitted version.

## Conflict of interest

The authors declare that the research was conducted in the absence of any commercial or financial relationships that could be construed as a potential conflict of interest.

## Publisher's note

All claims expressed in this article are solely those of the authors and do not necessarily represent those of their affiliated organizations, or those of the publisher, the editors and the reviewers. Any product that may be evaluated in this article, or claim that may be made by its manufacturer, is not guaranteed or endorsed by the publisher.

## Abbreviations

MMIV-IV: Medical Information Mart for Intensive Care-IV
MHR: Mean Heart Rate
ICU: Intensive care unit
AF: Atrial Fibrillation
SOFA: Sequential Organ Failure Assessment score
SAPS II: Simplified Acute Physiology Score II
GCS: Glasgow Coma Scale
CVD: Cardiovascular disease
SD: Standard deviation
IQR: Interquartile range
HR: Hazard Ratio
NIHSS: National Institute of Health Stroke Scale
HRV: Heart rate variability.
