# Supplementary material for: Effect of mean heart rate on 30-day mortality in ischemic stroke with atrial fibrillation: Data from the MIMIC-IV database
Source: Front Neurol. 2022 Oct 31;13:1017849. doi: 10.3389/fneur.2022.1017849 (PMC9660328; doi:10.3389/fneur.2022.1017849)
Supplement: Supplementary file 1 [file Data_Sheet_1.docx]

# eFigures in the supplement

**eFigure1. Flowchart of the study cohort.**

**eFigures2. Association between mean heart rate and 30-day mortality according to baseline characteristics. Each stratiﬁcation adjusted for all the factors (gender, mean SpO2, mean glucose, weight, SOFA score, Charlson comorbidity index, SAPS II, age, GCS, Ventilation use, and vasoactive drug use) except the stratiﬁcation factor itself. CVD, Cardiovascular disease; SOFA, Sequential Organ Failure Assessment; HR, hazard ratio; CI, Confidence interval.**

**eFigures3. Kaplan-Meier Survival Curves for day 30 of patients with ischemic stroke ranked the first according to the diagnostic sequence. MHR, mean heart rate; bpm, beats per minute.**

# eTables in the supplement

**eTable1 Definition of ischemic stroke and atrial fibrillation.**

**eTable2 Hazard ratio and 95% CI of mean heart rate for 30-day mortality.**

**eTable3 Hazard ratio and 95% CI of heart rate parameters for 30-day mortality in patients with ischemic stroke ranked the first according to the diagnostic sequence.**

## eTable1 in the Supplement

**eTable1 Definition of ischemic stroke and atrial fibrillation.**

| **Disease** | **ICD version** | | **ICD code** | **Long Title** |
| --- | --- | --- | --- | --- |
| Ischemic stroke | 9 | | 4350 | Basilar artery syndrome |
|  | 9 | | 4351 | Vertebral artery syndrome |
|  | 9 | | 4352 | Subclavian steal syndrome |
|  | 9 | | 4353 | Vertebrobasilar artery syndrome |
|  | 9 | | 4371 | Other generalized ischemic cerebrovascular disease |
|  | 9 | | 34660 | Persistent migraine aura with cerebral infarction, without mention of intractable migraine without mention of status migrainosus |
|  | 9 | | 34661 | Persistent migraine aura with cerebral infarction, with intractable migraine, so stated, without mention of status migrainosus |
|  | 9 | | 34662 | Persistent migraine aura with cerebral infarction, without mention of intractable migraine with status migrainosus |
|  | 9 | | 34663 | Persistent migraine aura with cerebral infarction, with intractable migraine, so stated, with status migrainosus |
|  | 9 | | 43301 | Occlusion and stenosis of basilar artery with cerebral infarction |
|  | 9 | | 43311 | Occlusion and stenosis of carotid artery with cerebral infarction |
|  | 9 | | 43321 | Occlusion and stenosis of vertebral artery with cerebral infarction |
|  | 9 | | 43331 | Occlusion and stenosis of multiple and bilateral precerebral arteries with cerebral infarction |
|  | 9 | | 43381 | Occlusion and stenosis of other specified precerebral artery with cerebral infarction |
|  | 9 | | 43391 | Occlusion and stenosis of unspecified precerebral artery with cerebral infarction |
|  | 9 | | 43401 | Cerebral thrombosis with cerebral infarction |
|  | 9 | | 43411 | Cerebral embolism with cerebral infarction |
|  | 9 | | 43491 | Cerebral artery occlusion, unspecified with cerebral infarction |
|  | 10 | | G450 | Vertebro-basilar artery syndrome |
|  | 10 | | G451 | Carotid artery syndrome (hemispheric) |
|  | 10 | | G452 | Multiple and bilateral precerebral artery syndromes |
|  | 10 | | G460 | Middle cerebral artery syndrome |
|  | 10 | | G461 | Anterior cerebral artery syndrome |
|  | 10 | | G462 | Posterior cerebral artery syndrome |
|  | 10 | | G463 | Brain stem stroke syndrome |
|  | 10 | | G464 | Cerebellar stroke syndrome |
|  | 10 | | I6300 | Cerebral infarction due to thrombosis of unspecified precerebral artery |
|  | 10 | | I63011 | Cerebral infarction due to thrombosis of right vertebral artery |
|  | 10 | | I63012 | Cerebral infarction due to thrombosis of left vertebral artery |
|  | 10 | | I63013 | Cerebral infarction due to thrombosis of bilateral vertebral arteries |
|  | 10 | | I63019 | Cerebral infarction due to thrombosis of unspecified vertebral artery |
|  | 10 | | I6302 | Cerebral infarction due to thrombosis of basilar artery |
|  | 10 | | I63031 | Cerebral infarction due to thrombosis of right carotid artery |
|  | 10 | | I63032 | Cerebral infarction due to thrombosis of left carotid artery |
|  | 10 | | I63033 | Cerebral infarction due to thrombosis of bilateral carotid arteries |
|  | 10 | | I63039 | Cerebral infarction due to thrombosis of unspecified carotid artery |
|  | 10 | | I6309 | Cerebral infarction due to thrombosis of other precerebral artery |
|  | 10 | | I6310 | Cerebral infarction due to embolism of unspecified precerebral artery |
|  | 10 | | I63111 | Cerebral infarction due to embolism of right vertebral artery |
|  | 10 | | I63112 | Cerebral infarction due to embolism of left vertebral artery |
|  | 10 | | I63113 | Cerebral infarction due to embolism of bilateral vertebral arteries |
|  | 10 | | I63119 | Cerebral infarction due to embolism of unspecified vertebral artery |
|  | 10 | | I6312 | Cerebral infarction due to embolism of basilar artery |
|  | 10 | | I63131 | Cerebral infarction due to embolism of right carotid artery |
|  | 10 | | I63132 | Cerebral infarction due to embolism of left carotid artery |
|  | 10 | | I63133 | Cerebral infarction due to embolism of bilateral carotid arteries |
|  | 10 | | I63139 | Cerebral infarction due to embolism of unspecified carotid artery |
|  | 10 | | I6319 | Cerebral infarction due to embolism of other precerebral artery |
|  | 10 | | I6320 | Cerebral infarction due to unspecified occlusion or stenosis of unspecified precerebral arteries |
|  | 10 | | I63211 | Cerebral infarction due to unspecified occlusion or stenosis of right vertebral artery |
|  | 10 | | I63212 | Cerebral infarction due to unspecified occlusion or stenosis of left vertebral artery |
|  | 10 | | I63213 | Cerebral infarction due to unspecified occlusion or stenosis of bilateral vertebral arteries |
|  | 10 | | I63219 | Cerebral infarction due to unspecified occlusion or stenosis of unspecified vertebral artery |
|  | 10 | | I6322 | Cerebral infarction due to unspecified occlusion or stenosis of basilar artery |
|  | 10 | | I63231 | Cerebral infarction due to unspecified occlusion or stenosis of right carotid arteries |
|  | 10 | | I63232 | Cerebral infarction due to unspecified occlusion or stenosis of left carotid arteries |
|  | 10 | | I63233 | Cerebral infarction due to unspecified occlusion or stenosis of bilateral carotid arteries |
|  | 10 | | I63239 | Cerebral infarction due to unspecified occlusion or stenosis of unspecified carotid artery |
|  | 10 | | I6329 | Cerebral infarction due to unspecified occlusion or stenosis of other precerebral arteries |
|  | 10 | | I6330 | Cerebral infarction due to thrombosis of unspecified cerebral artery |
|  | 10 | | I63311 | Cerebral infarction due to thrombosis of right middle cerebral artery |
|  | 10 | | I63312 | Cerebral infarction due to thrombosis of left middle cerebral artery |
|  | 10 | | I63313 | Cerebral infarction due to thrombosis of bilateral middle cerebral arteries |
|  | 10 | | I63319 | Cerebral infarction due to thrombosis of unspecified middle cerebral artery |
|  | 10 | | I63321 | Cerebral infarction due to thrombosis of right anterior cerebral artery |
|  | 10 | | I63322 | Cerebral infarction due to thrombosis of left anterior cerebral artery |
|  | 10 | | I63323 | Cerebral infarction due to thrombosis of bilateral anterior cerebral arteries |
|  | 10 | | I63329 | Cerebral infarction due to thrombosis of unspecified anterior cerebral artery |
|  | 10 | | I63331 | Cerebral infarction due to thrombosis of right posterior cerebral artery |
|  | 10 | | I63332 | Cerebral infarction due to thrombosis of left posterior cerebral artery |
|  | 10 | | I63333 | Cerebral infarction due to thrombosis of bilateral posterior cerebral arteries |
|  | 10 | | I63339 | Cerebral infarction due to thrombosis of unspecified posterior cerebral artery |
|  | 10 | | I63341 | Cerebral infarction due to thrombosis of right cerebellar artery |
|  | 10 | | I63342 | Cerebral infarction due to thrombosis of left cerebellar artery |
|  | 10 | | I63343 | Cerebral infarction due to thrombosis of bilateral cerebellar arteries |
|  | 10 | | I63349 | Cerebral infarction due to thrombosis of unspecified cerebellar artery |
|  | 10 | | I6339 | Cerebral infarction due to thrombosis of other cerebral artery |
|  | 10 | | I6340 | Cerebral infarction due to embolism of unspecified cerebral artery |
|  | 10 | | I63411 | Cerebral infarction due to embolism of right middle cerebral artery |
|  | 10 | | I63412 | Cerebral infarction due to embolism of left middle cerebral artery |
|  | 10 | | I63413 | Cerebral infarction due to embolism of bilateral middle cerebral arteries |
|  | 10 | | I63419 | Cerebral infarction due to embolism of unspecified middle cerebral artery |
|  | 10 | | I63421 | Cerebral infarction due to embolism of right anterior cerebral artery |
|  | 10 | | I63422 | Cerebral infarction due to embolism of left anterior cerebral artery |
|  | 10 | | I63423 | Cerebral infarction due to embolism of bilateral anterior cerebral arteries |
|  | 10 | | I63429 | Cerebral infarction due to embolism of unspecified anterior cerebral artery |
|  | 10 | | I63431 | Cerebral infarction due to embolism of right posterior cerebral artery |
|  | 10 | | I63432 | Cerebral infarction due to embolism of left posterior cerebral artery |
|  | 10 | | I63433 | Cerebral infarction due to embolism of bilateral posterior cerebral arteries |
|  | 10 | | I63439 | Cerebral infarction due to embolism of unspecified posterior cerebral artery |
|  | 10 | | I63441 | Cerebral infarction due to embolism of right cerebellar artery |
|  | 10 | | I63442 | Cerebral infarction due to embolism of left cerebellar artery |
|  | 10 | | I63443 | Cerebral infarction due to embolism of bilateral cerebellar arteries |
|  | 10 | | I63449 | Cerebral infarction due to embolism of unspecified cerebellar artery |
|  | 10 | | I6349 | Cerebral infarction due to embolism of other cerebral artery |
|  | 10 | | I6350 | Cerebral infarction due to unspecified occlusion or stenosis of unspecified cerebral artery |
|  | 10 | | I63511 | Cerebral infarction due to unspecified occlusion or stenosis of right middle cerebral artery |
|  | 10 | | I63512 | Cerebral infarction due to unspecified occlusion or stenosis of left middle cerebral artery |
|  | 10 | | I63513 | Cerebral infarction due to unspecified occlusion or stenosis of bilateral middle cerebral arteries |
|  | 10 | | I63519 | Cerebral infarction due to unspecified occlusion or stenosis of unspecified middle cerebral artery |
|  | 10 | | I63521 | Cerebral infarction due to unspecified occlusion or stenosis of right anterior cerebral artery |
|  | 10 | | I63522 | Cerebral infarction due to unspecified occlusion or stenosis of left anterior cerebral artery |
|  | 10 | | I63523 | Cerebral infarction due to unspecified occlusion or stenosis of bilateral anterior cerebral arteries |
|  | 10 | | I63529 | Cerebral infarction due to unspecified occlusion or stenosis of unspecified anterior cerebral artery |
|  | 10 | | I63531 | Cerebral infarction due to unspecified occlusion or stenosis of right posterior cerebral artery |
|  | 10 | | I63532 | Cerebral infarction due to unspecified occlusion or stenosis of left posterior cerebral artery |
|  | 10 | | I63533 | Cerebral infarction due to unspecified occlusion or stenosis of bilateral posterior cerebral arteries |
|  | 10 | | I63539 | Cerebral infarction due to unspecified occlusion or stenosis of unspecified posterior cerebral artery |
|  | 10 | | I63541 | Cerebral infarction due to unspecified occlusion or stenosis of right cerebellar artery |
|  | 10 | | I63542 | Cerebral infarction due to unspecified occlusion or stenosis of left cerebellar artery |
|  | 10 | | I63543 | Cerebral infarction due to unspecified occlusion or stenosis of bilateral cerebellar arteries |
|  | 10 | | I63549 | Cerebral infarction due to unspecified occlusion or stenosis of unspecified cerebellar artery |
|  | 10 | | I6359 | Cerebral infarction due to unspecified occlusion or stenosis of other cerebral artery |
|  | 10 | | I636 | Cerebral infarction due to cerebral venous thrombosis, nonpyogenic |
|  | 10 | | I6381 | Other cerebral infarction due to occlusion or stenosis of small artery |
|  | 10 | | I6389 | Other cerebral infarction |
|  | 10 | | I639 | Cerebral infarction, unspecified |
|  | 10 | | I6781 | Acute cerebrovascular insufficiency |
|  | 10 | | I6782 | Cerebral ischemia |
|  | 10 | | I67850 | Cerebral autosomal dominant arteriopathy with subcortical infarcts and leukoencephalopathy |
| Atrial fibrillation | | 9 | 42731 | Atrial fibrillation |
|  | | 10 | I48 | Atrial fibrillation and flutter |
|  | | 10 | I480 | Paroxysmal atrial fibrillation |
|  | | 10 | I481 | Persistent atrial fibrillation |
|  | | 10 | I4811 | Longstanding persistent atrial fibrillation |
|  | | 10 | I4819 | Other persistent atrial fibrillation |
|  | | 10 | I482 | Chronic atrial fibrillation |
|  | | 10 | I4820 | Chronic atrial fibrillation, unspecified |
|  | | 10 | I4821 | Permanent atrial fibrillation |
|  | | 10 | I489 | Unspecified atrial fibrillation and atrial flutter |
|  | | 10 | I4891 | Unspecified atrial fibrillation |

## eTable2 in the Supplement

| **eTable2 Hazard ratio and 95% CI of mean heart rate for 30-day mortality.** | | | | | | | | | | | |
| --- | --- | --- | --- | --- | --- | --- | --- | --- | --- | --- | --- |
| **Variable** | **Model1** | |  | **Model2^a^** | |  | **Model3^b^** | |  | **Model4^b^** | |
|  | **HR (95%CI)** | **P value** |  | **HR (95%CI)** | **P value** |  | **HR (95%CI)** | **P value** |  | **HR (95%CI)** | **P value** |
| **MHR per10, bpm** | 1.20 (1.11~1.3) | <0.001 |  | 1.21 (1.12~1.32) | <0.001 |  | 1.19 (1.09~1.31) | <0.001 |  | 1.20 (1.09~1.32) | <0.001 |
| **MHR tertials, bpm** | |  |  |  |  |  |  |  |  |  |  |
| <72 | 1.21 (0.78~1.87) | 0.403 |  | 1.22 (0.79~1.89) | 0.378 |  | 1.16 (0.69~1.93) | 0.578 |  | 1.17 (0.7~1.95) | 0.558 |
| 72~81 | 1(Reference) |  |  | 1(Reference) |  |  | 1(Reference) |  |  | 1(Reference) |  |
| >=82 | 1.92 (1.34~2.76) | <0.001 |  | 1.91 (1.32~2.75) | 0.001 |  | 1.80 (1.18~2.74) | 0.006 |  | 1.80 (1.18~2.75) | 0.007 |
| P for trend |  | 0.001 |  |  | 0.002 |  |  | 0.009 |  |  | 0.011 |
| MHR, mean heart rate; bpm, beats per minute, HR, hazard ratio; CI, Confidence interval; | | | | | | | | | | | |
| ^a^ There were 9 patients missing blood glucose data and 10 patients missing weight data, using sample size= 1384; | | | | | | | | | | | |
| ^b^ There were 9 patients missing blood glucose data,10 patients missing weight data, and 74 patients missing GCS data, using sample size= 1310; | | | | | | | | | | | |
| Model 1:no adjusted; | | | | | | | | | | | |
| Model 2: gender, mean SpO2, mean glucose, weight, Charlson comorbidity index, age; | | | | | | | | | | | |
| Model 3: gender, mean SpO2, mean glucose, weight, SOFA score, Charlson comorbidity index, SAPS II, age, GCS; | | | | | | | | | | | |
| Model 4: gender, mean SpO2, mean glucose, weight, SOFA score, Charlson comorbidity index, SAPS II, age, GCS, Ventilation use, vasoactive drugs use； | | | | | | | | | | | |
|  | | | | | | | | | | | |

## eTable3 in the Supplement

| **eTable3 Hazard ratio and 95% CI of heart rate parameters for 30-day mortality in patients with ischemic stroke ranked the first according to the diagnostic sequence.** | | | | | | | | | | | |
| --- | --- | --- | --- | --- | --- | --- | --- | --- | --- | --- | --- |
| **Variable** | **Model1** | |  | **Model2^a^** | |  | **Model3^a^** | |  | **Model4^a^** | |
|  | **HR (95%CI)** | **P value** |  | **HR (95%CI)** | **P value** |  | **HR (95%CI)** | **P value** |  | **HR (95%CI)** | **P value** |
| **MHR per10, bpm** | 1.21 (1.1~1.33) | <0.001 |  | 1.26 (1.13~1.4) | <0.001 |  | 1.23 (1.1~1.38) | <0.001 |  | 1.26 (1.12~1.41) | <0.001 |
| **MHR tertials, bpm** | |  |  |  |  |  |  |  |  |  |  |
| <72 | 0.94 (0.56~1.59) | 0.819 |  | 1.05 (0.62~1.78) | 0.853 |  | 1.12 (0.66~1.9) | 0.675 |  | 1.09 (0.64~1.86) | 0.740 |
| 72~81 | 1(Ref) |  |  | 1(Ref) |  |  | 1(Ref) |  |  | 1(Ref) |  |
| >=82 | 1.76 (1.15~2.69) | 0.010 |  | 1.96 (1.27~3.02) | 0.002 |  | 1.88 (1.22~2.91) | 0.004 |  | 1.96 (1.26~3.05) | 0.003 |
| P for trend | | 0.001 |  |  | 0.001 |  |  | 0.006 |  |  | 0.003 |
| MHR, mean heart rate; bpm, beats per minute, HR, hazard ratio; CI, Confidence interval; | | | | | | | | | | | |
| ^a^ There were 5 patients missing blood glucose data and 6 patients missing weight data, use sample size= 905; | | | | | | | | | | | |
| Model 1：non adjusted | | | | | | | | | | | |
| Model 2: gender, mean SpO2, mean glucose, weight, Charlson comorbidity index, age; | | | | | | | | | | | |
| Model 3: gender, mean SpO2, mean glucose, weight, SOFA score, Charlson comorbidity index, SAPS II, age, GCS; | | | | | | | | | | | |
| Model 4: gender, mean SpO2, mean glucose, weight, SOFA score, Charlson comorbidity index, SAPS II, age, GCS, Ventilation use, vasoactive drugs use； | | | | | | | | | | | |
