# Supplementary figures and images for: Effect of mean heart rate on 30-day mortality in ischemic stroke with atrial fibrillation: Data from the MIMIC-IV database
Source: Front Neurol. 2022 Oct 31;13:1017849. doi: 10.3389/fneur.2022.1017849 (PMC9660328; doi:10.3389/fneur.2022.1017849)

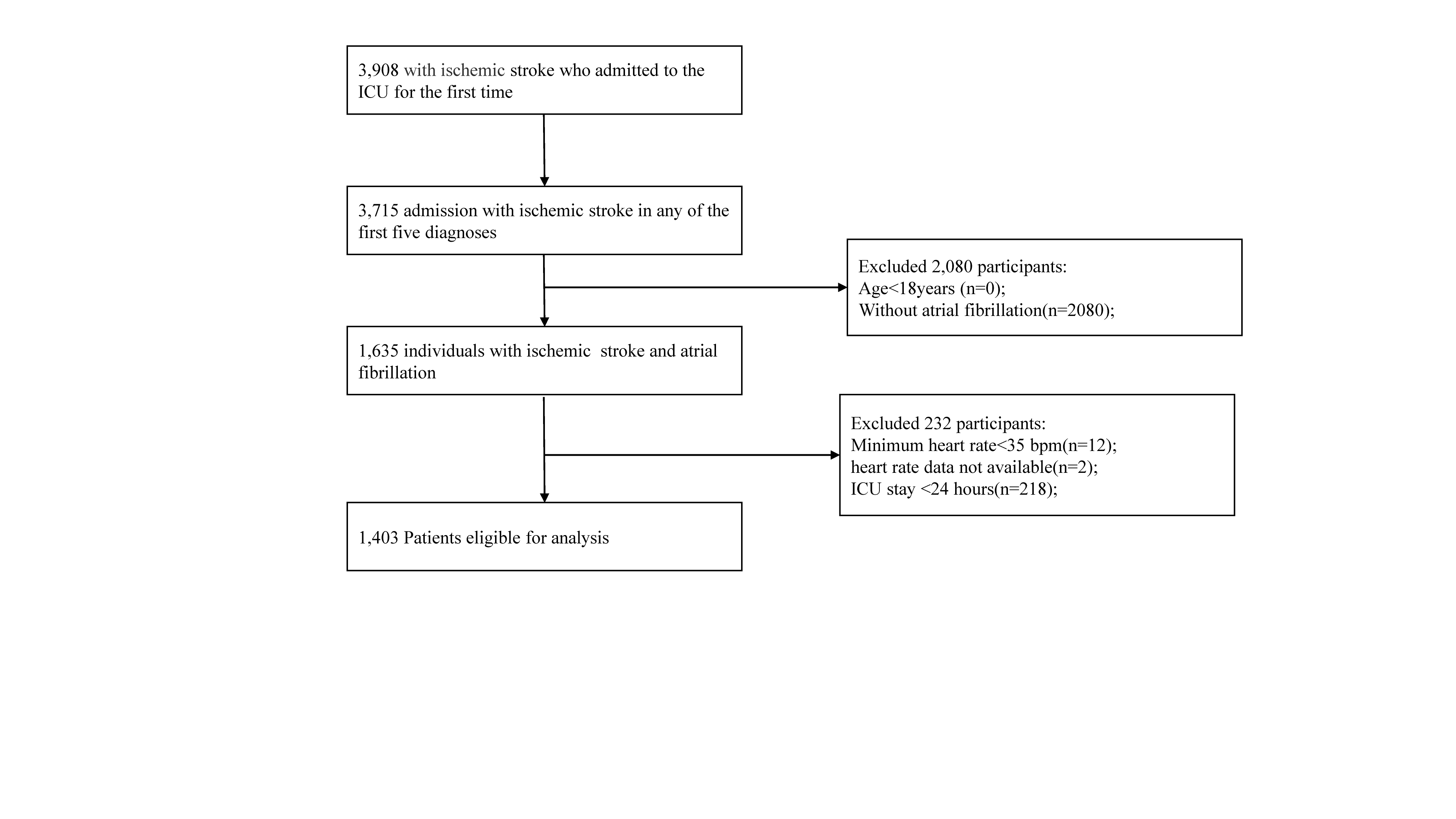

Supplement: Supplementary file 2 [file Image_1.TIF]

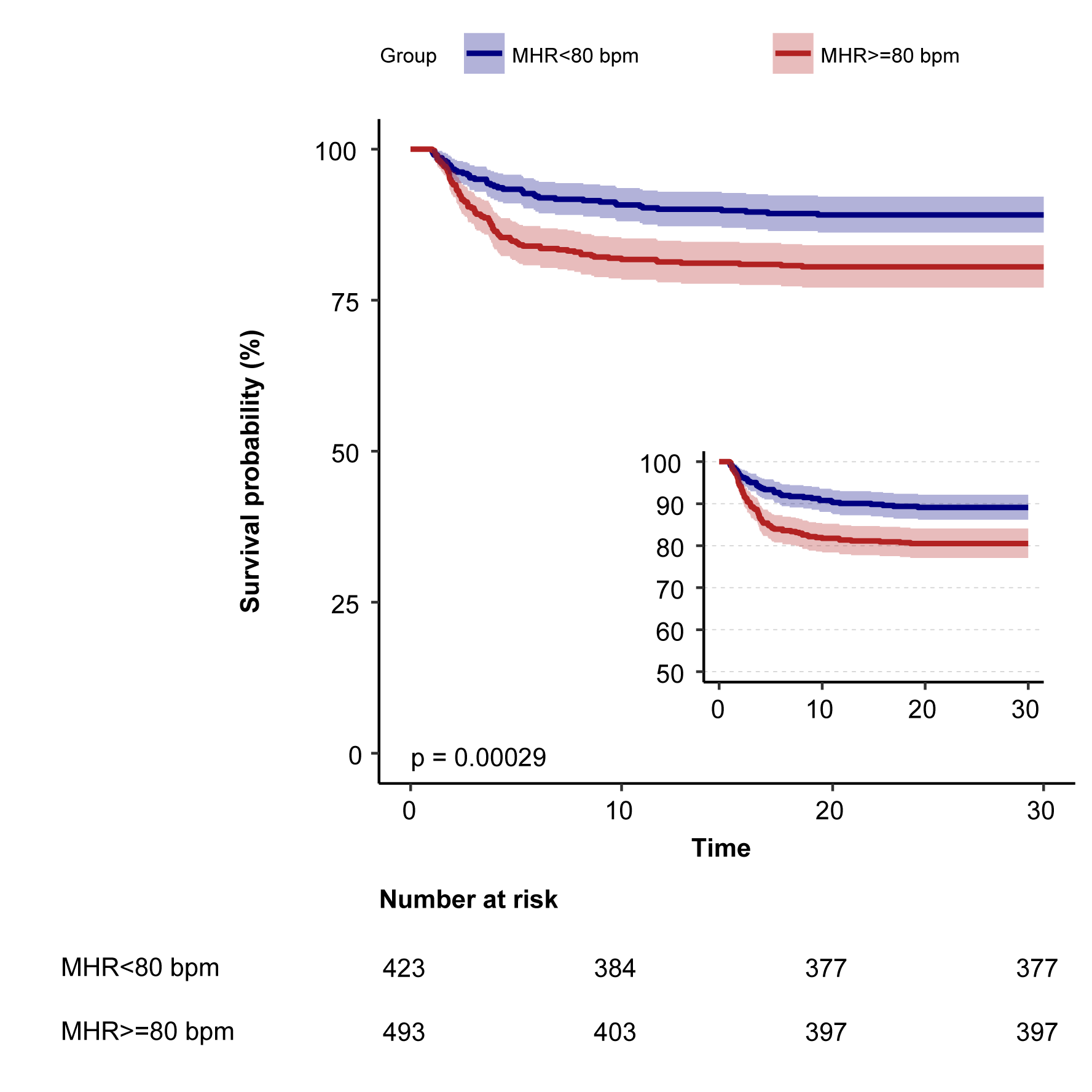

Supplement: Supplementary file 4 [file Image_3.TIF]
